# Do I Sound Sick? Condition‐Dependent Advertisement Signals in Naturally Infected Frogs

**DOI:** 10.1002/ece3.72350

**Published:** 2025-10-16

**Authors:** Trina L. Chou, Sarah A. R. Schrock, Mark Q. Wilber, Jessie C. Tanner

**Affiliations:** ^1^ Department of Ecology & Evolutionary Biology University of Tennessee Knoxville Tennessee USA; ^2^ Collaborative for Animal Behavior University of Tennessee Knoxville Tennessee USA; ^3^ School of Natural Resources University of Tennessee Institute of Agriculture Knoxville Tennessee USA; ^4^ Department of Psychology & Neuroscience University of Tennessee Knoxville Tennessee USA

**Keywords:** acoustic communication, *Batrachochytrium dendrobatidis* (*Bd*), behavioral tolerance, body condition, infection‐mediated signaling, spring peeper

## Abstract

Males of many frog and toad species advertise in leks. In these systems, female choice is based on male advertisement calls, which have been selected to convey information about an individual's quality. As such, calling behavior is an important aspect of reproductive fitness. Factors such as disease and infection can affect calling behavior, yet the direction and strength of these effects, as well as their underlying mechanisms, remain unclear. Calls are typically multicomponent displays, and traits within these displays can vary independently from one another both among and within individuals. It is important to understand the proximate infection‐imposed effects on signal production, as it allows us to make inferences about the downstream evolutionary consequences of such signaling. We studied the effects of *Batrachochytrium dendrobatidis* (*Bd*) infection on spring peeper ( *Pseudacris crucifer* ) advertisement calling behavior. We predicted that information about infection status would be present in dynamic traits (i.e., those that have high within‐individual variation) such that males with higher *Bd* infection loads would exhibit call traits less attractive to females. Overall, infection had little effect on male calling. There was no main effect of infection on call rate or dominant frequency. However, *Bd* infection did have body condition‐dependent effects on call duration, a trait with intermediate levels of within‐individual variation. As infection loads increased, males in better‐than‐average condition tended to have longer call durations, while males in worse‐than‐average conditions tended to have shorter call durations. Our results suggested that some males are more behaviorally tolerant than others when infected, meaning they invest more energy into current reproductive efforts (calling behavior) compared to future ones (fighting off disease). This may have interesting implications for the potential evolution of signals within these populations, as sexual selection for more attractive calls may also be selecting for more infected males.

## Graphical abstract

To understand whether infection has negative, sublethal effects on frog courtship behaviors, we conducted an observational field study of the effects of infection on spring peepers ( *Pseudacris crucifer* ). We found that increasing infection loads were associated with longer call durations in males in good condition, but were associated with shorter call durations in males in poor condition.

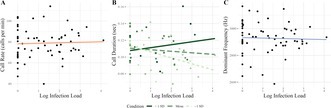

## 1 Introduction

Sexual selection drives the evolution of elaborate advertisement signals (reviewed in Rosenthal 2017). When making mating decisions, one sex (typically females) chooses mates based on these signals, which can be energetically costly to produce (Taigen et al. 1985; Stoddard and Salazar 2011). Brighter, longer, and otherwise more energy‐intensive signals generally indicate better mate quality (Zuk, Thornhill, Ligon, Johnson, Austad, et al. 1990; Hill 1991; Fischer et al. 2002). Theories of costly signaling suppose that the elaborate nature of these displays require energy expenditures that ensure signals reliably convey quality (Zahavi 1975; Stoddard and Salazar 2011). By choosing individuals with higher‐quality signals, receivers may benefit either directly (Grafe 1997; Siefferman 2003) or indirectly (Welch et al. 1998; Jaquiéry et al. 2010; Montoya and Torres 2015). In lek mating systems, receivers make mating decisions based primarily or entirely on advertisement signals produced by signalers (Höglund and Alatalo 1995). Signalers thus face fitness consequences if they cannot keep up with the energetic costs associated with signal production. These costs may vary from individual to individual based on differences in inherent physiological or metabolic processes (Taigen et al. 1985; Hill 2000). Additionally, individuals may differ in their ability to deal with increased energetic costs when faced with infection and disease, and these differences may impact signaling behaviors.

How infection mediates the production of advertisement signals, and subsequent selection through mate choice, remains poorly understood, as different studies have shown inconsistent outcomes (Beltran‐Bech and Richard 2014). When infected, individuals face direct trade‐offs between investing energy into either (1) current reproduction by maintaining or even increasing advertisement behaviors (behavioral tolerance), or (2) future reproduction by promoting behaviors oriented towards survival (behavioral resistance, see Stephenson and Adelman 2022). Infected individuals may increase immediate reproductive efforts when faced with pathogen‐induced mortality, leading them to express more ornamental displays (Clutton‐Brock 1984). This strategy as a form of behavioral tolerance could maximize lifetime reproductive success given that long‐term survival is unlikely (Foo et al. 2023). Alternatively, infection may prompt greater resource allocation towards bolstering immunological defenses, promoting strategies of behavioral resistance (Lochmiller and Deerenberg 2000). This resource allocation may come at the expense of investment in secondary sexual characteristics such as plumage coloration (Hõrak et al. 2004) or energy‐demanding behaviors such as acoustic signaling (Zuk, Thornhill, Ligon, and Johnson 1990). Infected individuals may be unable to maintain high‐performance signals, which may reduce the likelihood of attracting a mate. Over evolutionary time, males most resistant to infection may be the ones who display the most exaggerated ornaments (Hamilton and Zuk 1982; Folstad and Karter 1992; Balenger and Zuk 2014). Understanding the proximate effects of infection on signaling is important, given that the resulting signals may drive different evolutionary outcomes.

Empirical studies of infection‐mediated signaling in a variety of animal signaling systems have been repeatedly undertaken with varying results (Milinski and Bakker 1990; Madelaire et al. 2013; reviewed in Foo et al. 2023; Dougherty et al. 2023), and this lack of consensus may be driven by the complexity of the signals themselves. Signals are made up of multiple components that can independently evolve to carry different information, such as species identity, sex, social status, age, nutrition, and more (Hebets and Papaj 2005). For example, in American goldfinches ( *Carduelis tristis* ), black cap coloration provides information relevant to social interactions, while plumage and bill coloration provide information about infection status (McGraw and Hill 2000). While some information is relatively stable for a given individual over time (e.g., individual identity), other information may vary on short timescales (e.g., nutrition). Accordingly, signal components vary in their degree of within‐individual variation (Gerhardt 1991; Shaw and Herlihy 2000). Some traits are static (i.e., they vary little within individuals), making them useful indicators for unchanging information such as individual or species identity (Ewing 1989; Bailey 1991; Gerhardt 1991). Examples include pulse rate used in many frogs and crickets for species discrimination (Blair 1958; Loftus‐Hills and Littlejohn 1971; Pollack and Kim 2013) and carotenoid‐controlled patch size in birds during the annual molt (Badyaev et al. 2001; Van Dongen and Mulder 2008). Other traits are dynamic (i.e., they vary substantially within individuals) and are typically associated with energetic or other metabolic costs, such as call rate (Nally 1984; Hoback and Wagner 1997) or coloration brightness and pigmentation (Hill 1996, 2000). Dynamic traits are thus expected to convey honest information about short‐term changes in animal status, including those changes due to infection (reviewed in Dougherty et al. 2023).

Anuran amphibians (frogs and toads) are attractive models to test how infection affects different aspects of multicomponent advertisement signals. Male advertisement calls function to attract potential mates (Ryan 1988; Wells 2010). Calls comprise multiple traits that carry different information (Gerhardt 1991), and advertisement calling is an energetically demanding behavior (Taigen and Wells 1985; Prestwich 1994). Static traits such as pulse rate and dominant frequency are typically under stabilizing selection, while dynamic traits such as call rate and effort are typically under strong, directional, or threshold‐shaped sexual selection (Gerhardt 1991; Ryan and Keddy‐Hector 1992; Gerhardt and Brooks 2009; Tanner et al. 2017). Multiple studies (Table 1) have investigated the hypothesis that these dynamic traits should be sensitive to infection, as the energetic demands of calling could be further compounded by infection‐induced physiological stress. However, empirical evidence collected across frog taxa so far has been mixed: studies have reported positive, negative, and null effects of infection on dynamic call traits. Interestingly, while published studies have reported infection prevalence levels of between 18% and 100%, we note that the sublethal effects of infection may not be fully captured in the data because severely infected individuals may not produce advertisement signals at all.

**TABLE 1:** Review of the literature depicting how increasing infection loads affected specific calling traits. An upward‐facing arrow (↑) indicates an increase in the property (e.g., longer call, faster call rate). A downward‐facing arrow (↓) indicates a decrease in the property (e.g., shorter duration, slower rate). A dash (—) indicates no measured difference in that property. Gray cells indicate properties not measured.

Infection	Species	Study prevalence	Rate (dynamic)	Duration (dynamic)	Dominant frequency (static)	Pulse rate (static)	Source
Helminths	*Hyla versicolor*	62.20%	—	—			Hausfater et al. (1990)
Helminths	*Scaphiopus couchii*	26.23%		↑	—		Pfennig and Tinsley (2002)
Helminths	*Hypsiboas prasinus*	100%	↓	—	—		Madelaire et al. (2013)
Helminths	*Oophaga pumilio*	60.00%	—	—	—	↑	Pröhl et al. (2013)
Helminths	*Rhinella icterica*	96.40%	—				Moretti et al. (2017)
*Bd*	*Litoria rheocola*	59.02%	—	—	—	—	Greenspan et al. (2016)
*Bd*	*Hyla japonica*	21.43%	—	↑	—	↓	An and Waldman (2016)
*Bd*	*Pseudophryne pengilleyi*	40.00%		—	—	↑	Kelleher et al. (2021)
*Bd*	*Pseudacris regilla*	86.67%	—	↓	—	↑	Messersmith et al. (2024)
*Bd*	*Hyla japonica*	18.57%	—	—	—	—	Lee et al. (2025)

Of particular interest are the effects of the fungal pathogen *Batrachochytrium dendrobatidis* (*Bd*), the causative agent of the disease chytridiomycosis, which has led to the decline of hundreds of amphibian species worldwide (Scheele et al. 2019). *Bd* grows on amphibian skin and causes hyperkeratosis (skin thickening). This process disrupts normal physiological functioning and, in severe cases, can lead to cardiac arrest (Voyles et al. 2009). Although devastating to some populations, many species tolerate the pathogen, showing little or no known population‐level impacts. These varying responses are further reflected in reproductive behaviors, as some studies find that more infected males invest more in calling behaviors (An and Waldman 2016; Kelleher et al. 2021; Roznik et al. 2015) while others find that they invest less (Messersmith et al. 2024). Still others report no relationship between infection load and calling behaviors (Greenspan et al. 2016; Lee et al. 2025). Contrary to predictions of costly signaling theory, static traits such as pulse rate, rather than dynamic traits like call rate or call effort, were most commonly shown to be related to infection (Table 1). How populations adjust their reproductive behaviors in response to infection has important evolutionary implications; however, such non‐lethal effects are poorly understood in amphibian–*Bd* systems.

In the present study, we empirically investigated the advertisement calling behaviors of spring peepers ( *Pseudacris crucifer* ) in response to natural infections of *Bd*. Spring peepers are small chorus frogs (genus *Pseudacris* within the treefrog family *Hylidae*) commonly found in forested areas near temporary or semi‐permanent ponds in eastern North America. During their breeding season, males produce advertisement calls consisting of single‐toned “peeps”, often repeated up to 100 times per minute. In spring peepers, metabolic costs associated with calling are greater than metabolic costs incurred during locomotive behaviors (Taigen et al. 1985; Wells et al. 1996). Females prefer displays characterized by vigor (faster call rates, longer durations) and greater physical size (lower dominant frequencies), traits that signal higher mate quality (Lykens and Forester 1987; Sullivan and Hinshaw 1990; Wilhite 2014). Given *Bd’*s prevalence in wild populations (mean prevalence = 29.3%, 95% binomial CI = 22.5%–36.1%; from 2022 to 2024 in East Tennessee, Schrock and Wilber unpublished data) and the relatively low mortality rates associated with chytridiomycosis in this species (Gahl et al. 2012; Beyer et al. 2015), spring peepers provide an ideal system to investigate the sublethal effects of *Bd* infection on calling behavior. Additionally, they represent one of the first species with unpulsed calls to be studied in the context of *Bd* infection and calling. To quantify the potential fitness consequences of *Bd* infection on spring peeper calling, we recorded the advertisement calls of wild males and quantified *Bd* infection status and burden (load). We measured three call parameters (call rate, call duration, and dominant frequency) and classified each parameter based on its levels of within‐individual variation. We then assessed the relationships between these parameters and infection status and infection load. We predicted that information about infection would be present in dynamic traits as opposed to static or intermediate traits. We also predicted that males with higher *Bd* burdens may call less vigorously, potentially due to infection‐imposed energetic trade‐offs.

## 2 Materials and Methods

We collected data from 62 males at two locations in Knoxville, Tennessee. 30 males were from the largest pond at the Forks of the River Wildlife Management Area (35.92518, −83.853789, total perimeter ~510 m, total area ~7520 m^2^) and 32 males were from an urban pond known as Butterfly Lake (35.919945, −83.878892, total perimeter ~540 m, total area ~8240 m^2^). For each individual, we (1) recorded their calling behavior, (2) obtained general body measurements, and (3) swabbed their skin for *Bd*. All procedures were approved by the University of Tennessee's Institutional Animal Care and Use Committee under Protocol #2957‐0123 A. Scientific collection permits were granted by the Tennessee Wildlife Resource Agency.

### 2.1 Recording of Calling Behavior

We recorded individual males calling when chorus activity was strong (typically between 20:00 and 24:00 between March 3 and April 18, 2024). We used a DR‐40X Linear PCM recorder (sampling rate of 44.1 kHz, 16‐bit resolution; TASCAM, USA) connected to a directional MKE 600 shotgun microphone (Sennheiser, Germany) held at a distance of 1 m directly from the calling individual. We recorded a minimum of 2 min of vocal activity.

### 2.2 General Body Measurements

Animals were captured by hand and temporarily contained in a clean, transparent plastic sack. Snout‐to‐vent length (SVL) and tibia length (TL) were measured to the nearest 0.01 mm using dial calipers. Body mass was measured to the nearest 0.1 g using Pesola hanging spring scales. Prior to capture, body temperature was measured to the nearest 0.1°C with a dual laser infrared thermometer (factory calibrated and accurate to ±1.0°C, emissivity = 0.95; Fisherbrand, USA) aimed at the dorsum (back) of the frog.

### 2.3 Swabbing for *Bd*

Individuals were then swabbed for *Bd* using fine‐tip sterile swabs following a standardized protocol, making 20 strokes on the abdomen and thighs and an additional 20 strokes among the webbing of the foot (Hyatt et al. 2007). All swabbing was performed by the same individual (TLC) to reduce variation in technique associated with multiple individuals. Clean nitrile gloves were worn and changed between samples to prevent cross‐contamination. Swabs thus collected were stored individually in microcentrifuge tubes and then frozen at 4°C until molecular analysis. We avoided toe‐clipping for ethical reasons and instead reduced the probability of recapturing individuals by recording in separate areas of the ponds both within a night and across nights.

### 2.4 Molecular Analyses

We extracted DNA from the swabs using Qiagen DNEasy kits. We measured *Bd* fungal loads using TaqMan real‐time quantitative PCR (qPCR) as outlined in Boyle et al. (2004) with modifications by Hyatt et al. (2007). This process tags the ITS‐1 region of the *Bd* genome with a fluorescent probe and amplifies it. During amplification, the process is monitored in real‐time and the amount of fluorescence is measured and scaled to the amount of targeted DNA present. The cycle time at which fluorescence reaches a threshold is output as the quantitative metric (cycle threshold, or Ct, values), with lower values indicating the sample reached the threshold more quickly and, thus, contained more target DNA. A lab‐specific standard curve was used to estimate zoospore equivalents (ZE) based on Ct values, with samples containing ZEs ≥ 1 being considered “infected”. All samples were run in duplicate, and the average loads between these two runs were used in the analyses. In *N* = 3 cases, one replicate detected some level of *Bd* and the other had undetectable levels. We re‐ran our statistical analyses excluding these samples. Results were insensitive to the exclusion of these samples, thus all reported results include the full dataset.

### 2.5 Acoustical Analyses

For each male, 28–30 consecutive advertisement calls were analyzed using Raven Pro Software v 1.6.5 (Cornell Lab of Ornithology, Ithaca, NY, USA). Calls for analysis were chosen as consecutive calls starting from at least the sixth call of a bout to avoid irregularities in calling associated with the start and end of calling bouts. Using the “Band Limited Energy Detector” feature in Raven, calls were selected in the audio file and measured for three features: call rate, call duration, and dominant frequency. *Call rate* (calls per sec) was calculated as the inverse of call period (time from the onset of one call to the onset of the next), while *call duration* (in ms) was measured as the time between the onset and offset of a call. *Dominant frequency* (Hz) was measured as the frequency with the greatest relative amplitude from a power spectrum generated in Raven (Hann windows of 1024 points and 75% overlap).

### 2.6 Statistical Analyses

#### 2.6.1 Exploratory

We analyzed all data using RStudio running R version 4.4.0 (R Core Team 2024). Preliminary t‐tests were first run to determine differences between the two populations. Neither SVL (mean ± SD: 27.40 ± 1.72 mm) nor TL (mean ± SD: 13.90 ± 0.94 mm) differed between the two locations (*t*_60_ = 0.10, *p* = 0.92; *t*_58_ = 0.53, *p* = 0.60); however, males from Forks of the River weighed more (mean ± SD: 1.60 ± 0.31 g) than frogs from Butterfly Lake (mean ± SD: 1.44 ± 0.32 g; *t*_60_ = 2.09, *p* = 0.04). We calculated a body condition index (BCI) for each individual by measuring the residual from a linear regression of the cube root of mass on SVL divided by SVL (Baker 1992). No differences in call parameters, infection status, nor load between sites were significant at the *α* = 0.05 level; therefore, all further analyses considered the entire data set as one population (*N* = 62).

Spring peeper call parameters are correlated with body temperature and size, similar to other anurans (Forester and Czarnowsky 1985; Sullivan and Hinshaw 1990; Dye et al. 2024; Messersmith et al. 2024; see Figure S1). We therefore standardized all parameters of all calls (28–30 calls per male) first to a common temperature of 14°C, and then to an SVL of 27 mm, the standard temperature and SVL measurement used in studies involving the *Pseudacris* genus (Lykens and Forester 1987; Bee et al. 2010; Vélez and Guajardo 2021; Messersmith et al. 2024) following the methods of Platz and Forester (1988). In short, a regression line was fit for the standardizing parameter (temperature or SVL) against the call parameter (call rate, duration, or dominant frequency), the vertical difference (i.e., the residual) between each point and the line was observed, and that difference was used to quantify what the value would have been at the desired temperature. After the temperature and SVL correction were performed, we calculated coefficients of variation within each male's set of calls for each parameter by dividing the standard deviation of the signal set by the mean (CV_w_ = SD/mean). Coefficients of variation for the call parameters at the population level were then calculated as the average CV_w_ across all males measured. Depending on the CV_w_ value, call parameters were classified as static, dynamic, or intermediate using cutoff values established in the literature (Gerhardt 1991; Wollerman 1998; Gerhardt and Huber 2002): parameters with CV_w_ < 0.05 were labeled as static, parameters with CV_w_ > 0.05 and < 0.1 were labeled as intermediate, and parameters with CV_w_ > 0.1 were labeled as dynamic.

#### 2.6.2 Modeling

We first tested whether infection classification based on the combination of the call parameters was possible with a linear discriminant analysis (LDA). LDA maximizes the differences between known groups through a linear combination of the predictor variables, then makes classifications based on the values of the resulting discriminant function. We used the lda() function in the *MASS* package in R to classify a male's infection status (infected, uninfected) based on the combination of his three call parameters (call rate, call duration, dominant frequency). We then assessed whether the model correctly classified males' infection statuses by looking at the confusion matrix generated by the LDA model. To assess the relationship between infection load and each of the call parameters individually, we created one model for each calling parameter as the dependent variable, resulting in three linear regression models. For all models, independent variables included the fixed effects of log‐transformed ZE, BCI, and the interaction between the two. The interaction was included because in at least one species of rainforest frog (*Litoria rheocoloa*), body condition interacts with infection to affect calling (Roznik et al. 2015). We tested for significance (Bonferroni correction, *α* = 0.05/3 = 0.017) using a type‐III analysis‐of‐variance test using the Anova() function in the *car* package and extracted partial *η* ^2^ effect sizes using the eta_squared() function in the *effect size* package.

## 3 Results

**FIGURE 1:**
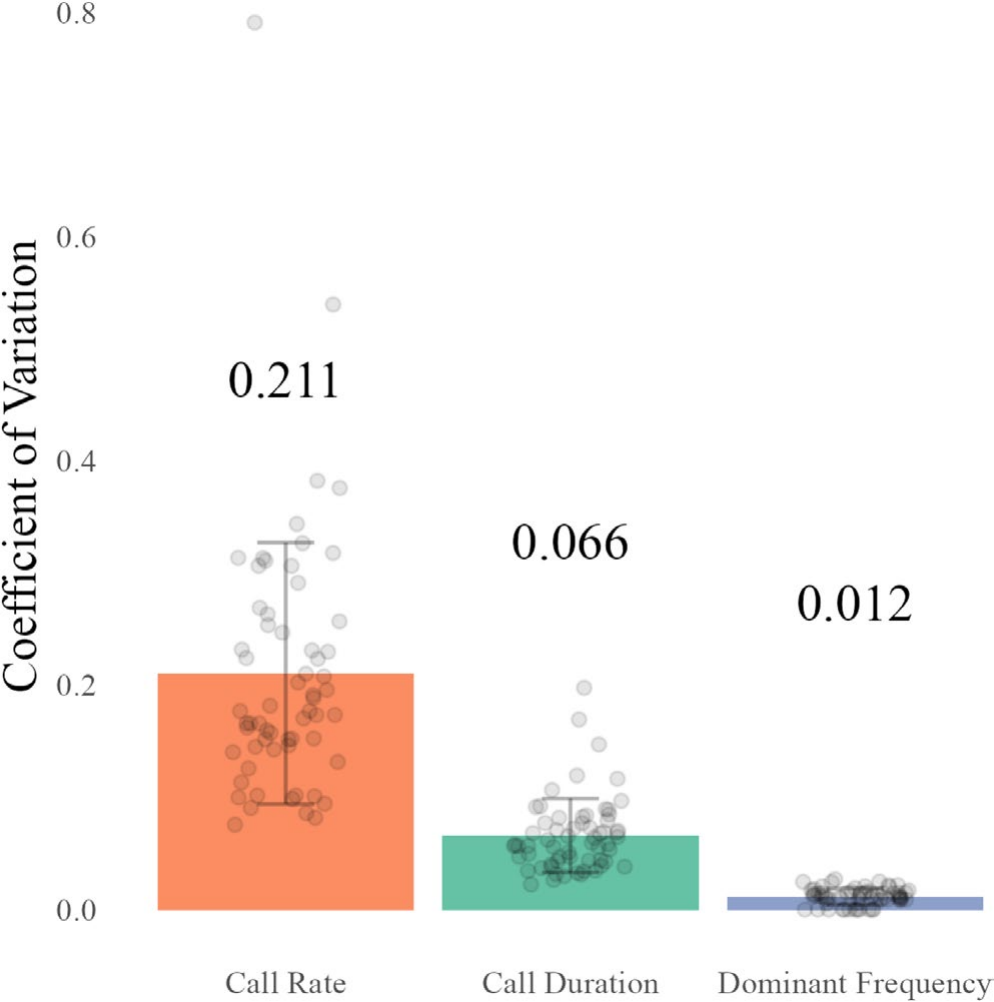
Based on their average coefficients of variation within males (CV_w_), call traits were classified as static (CV_w_ < 0.05), intermediate (0.05 < CV_w_ < 0.1), or dynamic (CV_w_ > 0.1). Dominant frequency was classified as static, call duration as intermediate, and call rate as dynamic. Error bars represent standard deviations of the mean, and individual data points represent each male's CV_w_ (*N* = 62).

In this population of spring peepers, dominant frequency varied relatively little within individuals (mean ± SD: CV_w_ = 0.012 ± 0.007) and was therefore classified as a static property. Call duration was classified as an intermediate property (CV_w_ = 0.066 ± 0.033). Call rate was variable within individuals (CV_w_ = 0.211 ± 0.117) and was classified as a dynamic property. Variation in dominant frequency across individuals was relatively similar, while variation in call rate varied widely from male to male (Figure 1).

**FIGURE 2:**
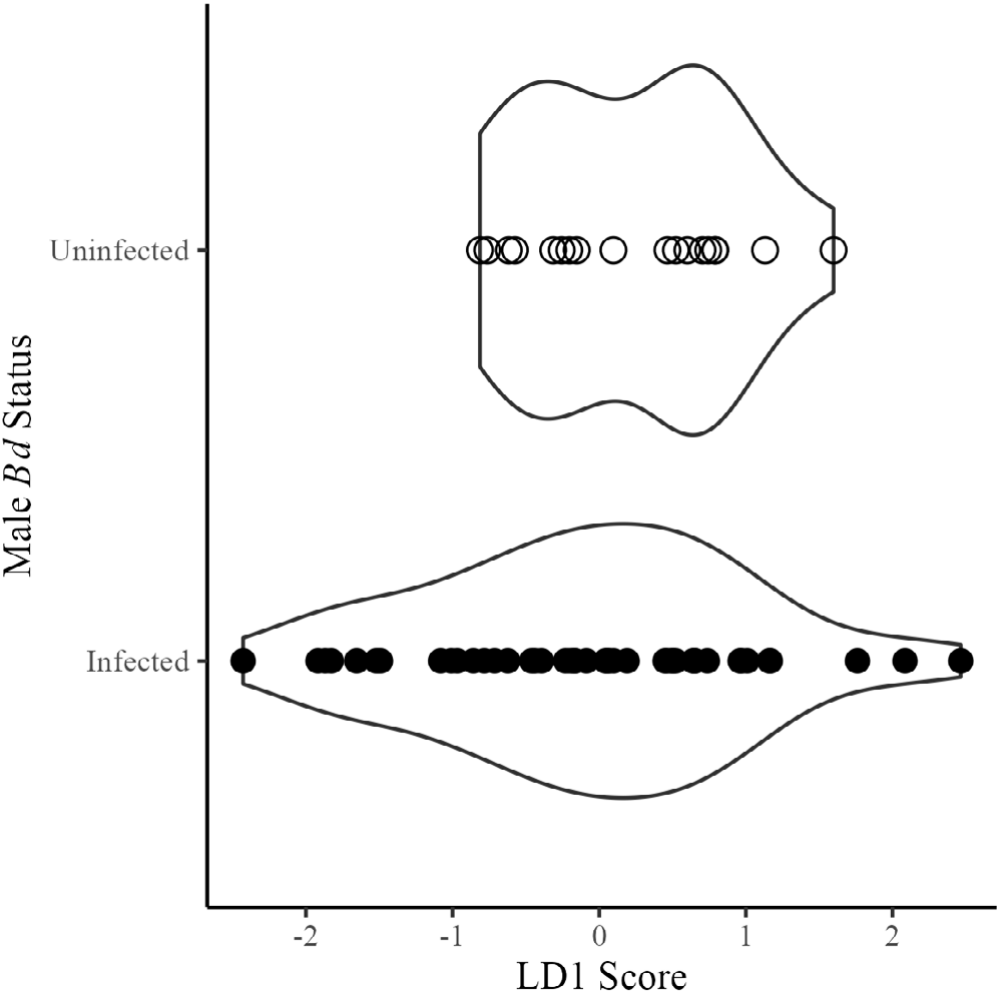
Males (*N* = 62) could not be separated by infection status (black = infected; white = uninfected) using a linear discriminant function based on the three call parameters. The linear discriminant score (LD1) was primarily affected by call duration.

**FIGURE 3:**
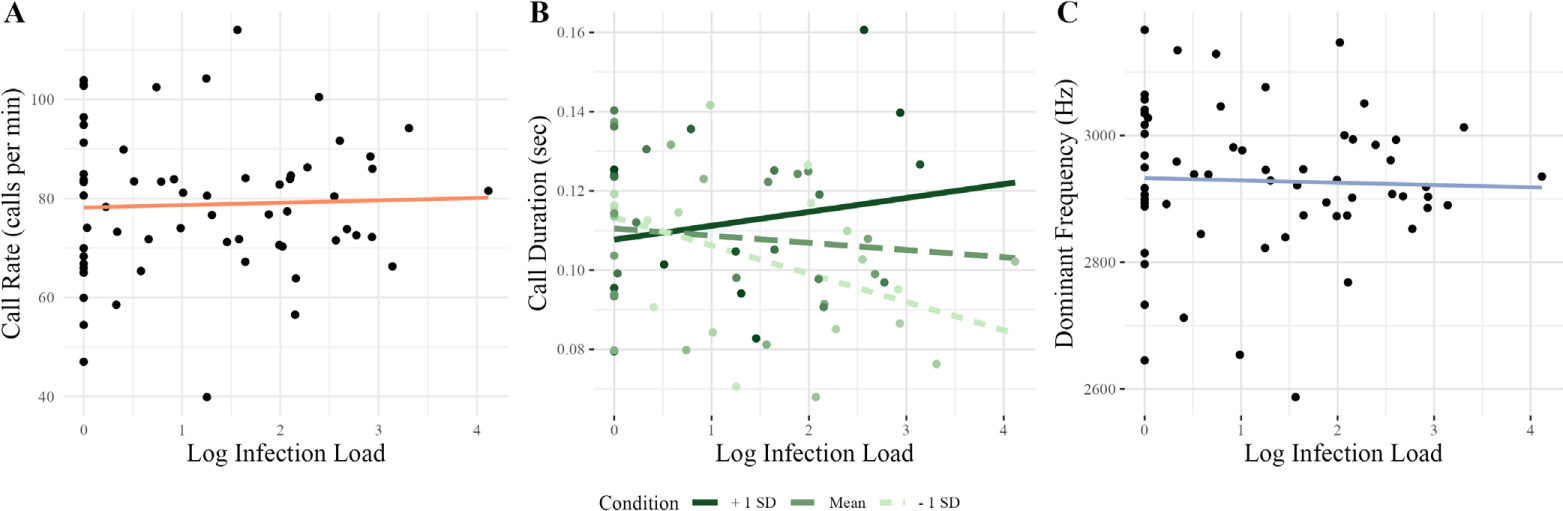
Plots of Log ZE infection load against (A) call rate (calls per min), (B) call duration (sec), and (C) dominant frequency (Hz). For call duration, the interaction between infection load and body condition was marginally significant with a Bonferroni correction, with males in better condition having longer call durations as infection load increases, while males in poor condition had shorter call durations as infection load increased. Slope lines depicted in (B) have been generated for the purposes of visualization and were not tested categorically in the models (*N* = 62).

Most (70.97%) males were infected with *Bd* to some degree. For infected males, fungal load values had a mean of 528 ZE ranging from 1 to 13,095 ZE with a median of 87 ZE. The linear discriminant function based on the three call parameters was heavily loaded by call duration (*β* = 52.39 compared to *β* = 0.03 for call rate and *β* = 0.004 for dominant frequency); however, calls were not discriminable based on their infection status, as all individuals were classified by the model as infected (Figure 2). The interaction between fungal load and body condition on call duration had a medium effect size (*η* ^2^ = 0.09) and was marginally significant with the Bonferroni correction (*F*_1, 58_ = 5.952, *p* = 0.018; Table 2). For males with BCI values greater than zero (“good” condition), increasing infection loads were correlated with longer call durations. The reverse was true for males with BCI values less than zero (“poor” condition), as increasing infection loads correlated with shorter call durations (Figure 3B). Neither *Bd* fungal load, body condition, nor the interaction between fungal load and body condition significantly affected either call rate or dominant frequency (Figure 3, Table 2).

**TABLE 2:** Results from analysis of variance of the three linear regression models for each of the call traits. Marginally significant predictor variables are italicized (*α* = 0.017). Partial η^2^ effect sizes are classified as small (~0.01), medium (~0.06), or large (> 0.14). For call duration, the interaction between infection load and condition was marginally significant, with a medium effect size. No other variables were significant.

Predictor variable	*F*_1, 58_	Pr(> *F*)	*η* ^2^
Call rate			
Log infection	0.077	0.782	< 0.01
Condition	0.670	0.417	0.01
Log infection × Condition	0.805	0.373	0.01
Call duration			
Log infection	0.699	0.407	0.01
Condition	0.480	0.491	< 0.01
*Log infection × Condition*	*5.952*	*0.018*	*0.09*
Dominant frequency			
Log infection	0.112	0.739	< 0.01
Condition	0.226	0.636	< 0.01
Log infection × Condition	0.068	0.796	< 0.01

## 4 Discussion

Here, we collected acoustic and molecular data to better understand how *Bd* infection affects signaling behavior in spring peepers. Because the evidence across anuran taxa has provided conflicting results while examining disparate call parameters (Table 1), more research is needed across amphibian species and infection types to understand when and how infection affects sexual signaling. Our own empirical investigation found that males could not be classified as infected or uninfected based on their sexual signals alone. Additionally, infection overall had little effect on call traits. Infection intensity was not associated with differences in call rate or dominant frequency, but there was a marginally significant interaction (using a strict Bonferroni correction) between call duration and body condition that points towards condition‐dependent signaling. Males in “good” condition had longer calling durations as infection load increased, while males in “poor” condition had shorter calling durations. This partially supported our second prediction that males with higher *Bd* infection intensity would call less vigorously, potentially due to energetic trade‐offs. However, it also suggests that infection's effects may be dependent on some intrinsic factors of individuals. Given that females display general preferences for longer call durations (Gerhardt et al. 2000; Wilhite 2014), infected males in “good” condition may display competitive advantages compared to both uninfected males and infected males in “poor” condition. In summary, we found that infection had a complex effect on the spring peeper's multicomponent advertisement call.

Although sexual selection theory predicts that infection would affect dynamic traits as opposed to static ones due to the variability of dynamic traits on short timescales (Gerhardt and Huber 2002; Maynard Smith and Harper 2003; Dougherty et al. 2023), our findings did not support this prediction. Rather, varying infection levels in spring peepers were correlated with differences in call duration, of which measured within‐individual variation did not meet the threshold to classify it as a dynamic trait. This finding aligns with previous studies both in anurans (An and Waldman 2016; Kelleher et al. 2021; Messersmith et al. 2024) and non‐anuran species (male guppies, *Poecilia reticulata* ; Stephenson et al. 2020; wolf spiders, *Schizocosa ocreata* ; Gilbert and Uetz 2016) that commonly demonstrate changes in non‐dynamic traits in response to increasing infection load. Given that several species such as *Pseudacris maculata, Pseudacris regilla*, and *Pseudacris feriarum* use pulse rates and call durations for species identification (Straughan 1975; Platz 1989; Bush et al. 2002; Lemmon 2009), infection‐induced changes in these traits may interfere with species recognition mechanisms and alter mating outcomes. The surprising influence of infection on non‐dynamic signal traits may partially be influenced by other aspects of the mating environment that aren't signal feature‐dependent, such as nightly chorus attendance, which may be the best predictor of male mating success across multiple species of frogs (Gerhardt et al. 1987; Murphy 1994; Friedl and Klump 2005). Despite the prevalence of multicomponent signals, few studies have specifically examined how static or dynamic traits might be differentially affected by external factors such as infection.

Infected males in good condition had longer calling durations, appearing to invest more into behavioral tolerance (immediate reproduction) as opposed to behavioral resistance (future reproduction). Other studies have similarly found positive associations between infection load and static traits in anuran signaling systems (Pröhl et al. 2013; An and Waldman 2016; Kelleher et al. 2021; Messersmith et al. 2024), contrary to predictions made by sexual selection theory. Pulse rate in particular seems to be affected by variation in infection (Table 1), despite pulse rate typically being a static property that varies little within individuals over time (Gerhardt 1991; Shaw and Herlihy 2000). It is unclear why pulse rate should change with *Bd* infection; however, a possible explanation could be due to associated energy expenditure. For species of frogs with unpulsed advertisement calls such as spring peepers, each call is made with a single contraction of the trunk muscles (Pough et al. 1992). Thus, calls with longer call durations are more energetically expensive to produce than shorter ones when controlling for call rate (Ophir et al. 2010). For species with pulsed calls, however, pulses are generated through cycles of contraction and relaxation of the trunk muscles (Girgenrath and Marsh 1997). Thus, increased pulse rates when controlling for call rate may also lead to increases in energy expenditure. Increases in these traits may thus be seen as investment in behavioral tolerance, despite typically being non‐dynamic traits. Explicitly considering the patterns of within‐individual variation in signal traits may help us better explain observed differences in effects across species. Future studies should explicitly consider how call complexity interacts with factors such as pathogen infection and intensity to affect signaling behavior.

Our observation that males in good condition exhibit behavioral tolerance may be explained by their regular exposure to *Bd* within our population. *Bd* persists endemically in these study areas, and while there is evidence for acquired immunity to *Bd* in some anurans (McMahon et al. 2014; Waddle et al. 2024), this resistance is often temporary and does not prevent *Bd* infection. Given these high levels of exposure and prolonged infections, a response based on behavioral resistance would be evolutionarily costly: calling parameters would be consistently repressed given re‐exposures to *Bd*, altering the attractiveness of their signals and thus reducing reproductive potential. Future studies should examine the interplay between acquired immunity and trade‐offs between utilizing behavioral resistance vs. tolerance strategies in endemic host–parasite systems, as it might be predicted that stronger immunity might lead to the emergence of behavioral resistance strategies.

In this present study, we found that males in poor condition had shortened call durations as infection intensities increased. That is, they appeared to decrease investment in current reproduction, perhaps reflecting a trade‐off between investment of energy into calling versus resisting infection. Condition‐dependent calling was also documented in the mistfrog *Litoria rhecoloa* in Australia. Infected *L. rhecoloa* males in good condition were ~30% more likely to call than uninfected males, while infected males in poor condition were ~40% less likely to call than uninfected males (Roznik et al. 2015). Males in good condition may be more behaviorally tolerant than males in poor condition and suffer less from the energetic costs of infection. These pre‐existing differences among males thus seem to be exacerbated by infection, allowing only individuals in good condition to respond appropriately. Because we sampled only calling males, we note the possibility that some males might be so infected with *Bd*—or suffering from chytridiomycosis—as to be unable to call. If males with extremely high *Bd* loads do not call, we may have a dataset that does not capture the highest levels of infection intensity observed in this species. Despite this potential limitation, however, we still observed differences in call durations at the intensity levels we measured. Future work should experimentally infect and clear individuals with *Bd* to understand the true causal effects of infection on individual calling behavior.

Spring peepers have low levels of mortality attributable to *Bd* infection in the wild, yet we found that *Bd* led to changes in their calling behavior. Given that advertisement calls heavily influence mating outcomes and thus potential future fitness, infection may have negative, sublethal impacts that reduce an individual's fitness in ways not captured by mortality alone. In spring peepers and many other species of frogs, females are capable of discriminating among males on the basis of fine‐scale temporal and spectral properties and show preferences for longer call durations (Klump and Gerhardt 1987; Schwartz et al. 2001; Tárano and Herrera 2003; Bee 2008; Wilhite 2014). Our data therefore suggest that infected males in good condition may increase their chances of attracting a female compared to both uninfected males and infected males in poor condition. This may lead to outcomes where females repeatedly choose to mate with males who are more infected, minimizing population‐level selection for disease resistance but potentially maximizing selection for disease tolerance. However, whether changes in calling behavior change female behavior depends on whether females discriminate among calls based on extant variation. In our study, the predicted difference in call duration between males who are infected is small, on the order of approximately 40 ms. Previous research on spring peepers has documented female discrimination on the basis of call duration differences as small as 75 ms (Doherty and Gerhardt 1984); however, smaller changes, on the order of the effect we detected, have not been tested. To truly understand the impacts of this infection‐mediated change in signals on the potential evolutionary trajectory of these signals, we would need to conduct mate‐choice experiments with females to empirically test whether receivers discriminate between prospective mates based on our observed differences in call duration.

In conclusion, our results add to the growing literature detailing the effects of infection on anuran signaling, an important determinant of amphibian reproductive success. Infection intensities affected spring peeper calling in a condition‐dependent manner by influencing a non‐dynamic trait. These studies are important because for species that attract mates based on their communication signals, infection may not only directly affect their fitness through mortality but also indirectly through the alteration of their mating signals. Understanding how information about infection status is expressed through amphibian calling can help us better predict how populations will respond over time to infection. Additionally, we found that even among the relatively limited research conducted so far, there exists variation in the ways in which species, call traits, and even individuals are affected by and respond to infection. More research is needed to understand what factors are driving this variation across the multiple levels, such as life history, call trait complexity, and even species' local relationships to infections.

## Author Contributions

**Trina L. Chou:** conceptualization (equal), data curation (lead), formal analysis (lead), investigation (lead), methodology (lead), supervision (equal), visualization (lead), writing – original draft (lead). **Sarah A. R. Schrock:** formal analysis (equal), investigation (supporting), methodology (supporting), writing – original draft (supporting), writing – review and editing (equal). **Mark Q. Wilber:** conceptualization (equal), resources (equal), writing – review and editing (equal). **Jessie C. Tanner:** conceptualization (equal), data curation (supporting), investigation (supporting), resources (equal), supervision (supporting), writing – review and editing (equal).

## Conflicts of Interest

The authors declare no conflicts of interest.

## Supporting information


**Data S1:** ece372350‐sup‐0001‐DataS1.zip.

## Data Availability

All data and code are available as Supporting Information.
