# Supplementary figures and images for: Do I Sound Sick? Condition‐Dependent Advertisement Signals in Naturally Infected Frogs
Source: Ecol Evol. 2025 Oct 16;15(10):e72350. doi: 10.1002/ece3.72350 (PMC12529013; doi:10.1002/ece3.72350)

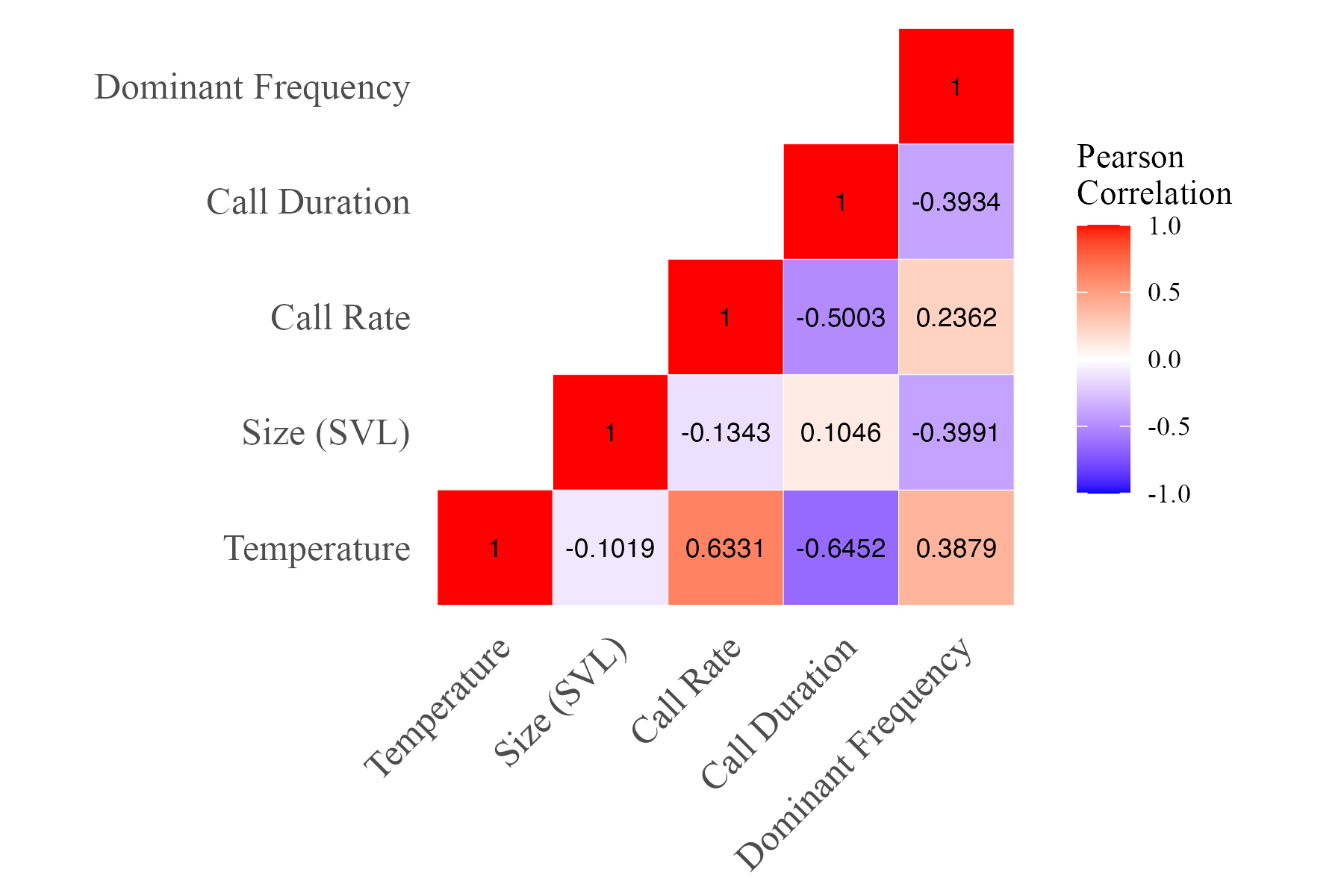

Supplement: Supplementary file 1 — Data S1: ece372350‐sup‐0001‐DataS1.zip. [file ECE3-15-e72350-s001.zip › ECE3_72350_f1_suppfig1.tiff]
